# Genome-Wide Identification and Characterization Analysis of WUSCHEL-Related Homeobox Family in Melon (*Cucumis melo* L.)

**DOI:** 10.3390/ijms241512326

**Published:** 2023-08-01

**Authors:** Lingli Tang, Yuhua He, Bin Liu, Yongyang Xu, Guangwei Zhao

**Affiliations:** 1 Zhengzhou Fruit Research Institute, Chinese Academy of Agricultural Sciences, Zhengzhou 450009, China; tanglingli@caas.cn (L.T.); heyuhua@caas.cn (Y.H.); 2 National Nanfan Research Institute, Chinese Academy of Agricultural Sciences, Sanya 572000, China; 3 Hami-melon Research Center, Xinjiang Academy of Agricultural Sciences, Urumqi 830091, China; liu.bin@xaas.ac.cn

**Keywords:** melon, WUSCHEL-related homeobox, phylogenetic analysis, abiotic stresses, gene expression

## Abstract

WUSCHEL-related homeobox (WOX) proteins are very important in controlling plant development and stress responses. However, the WOX family members and their role in response to abiotic stresses are largely unknown in melon (*Cucumis melo* L.). In this study, 11 WOX (CmWOX) transcript factors with conserved WUS and homeobox motif were identified and characterized, and subdivided into modern clade, ancient clade and intermediate clade based on bioinformatic and phylogenetic analysis. Evolutionary analysis revealed that the CmWOX family showed protein variations in Arabidopsis, tomato, cucumber, melon and rice. Alignment of protein sequences uncovered that all CmWOXs had the typical homeodomain, which consisted of conserved amino acids. *Cis*-element analysis showed that *CmWOX* genes may response to abiotic stress. RNA-seq and qRT-PCR results further revealed that the expression of partially *CmWOX* genes are associated with cold and drought. *CmWOX13a* and *CmWOX13b* were constitutively expressed under abiotic stresses, *CmWOX4* may play a role in abiotic processes during plant development. Taken together, this study offers new perspectives on the CmWOX family’s interaction and provides the framework for research on the molecular functions of *CmWOX* genes.

## 1. Introduction

WUSCHEL-related homeobox (WOX) protein family is one of the subclades of the plants-specific homeodomain (HD) superfamily. The family members are distinguished through a conserved HD, which is typically formed of 60–66 amino acids with a specific structure. The HD domain, which is highly conserved among WOX protein families and essential to their functions, can bind particular DNA sequence to control the expression of downstream genes [1,2,3].

Plant evolution has led to a progressive expansion of the WOX family, for example, *Physcomitrella patens* possess three WOX, whereas unicellular green algae only have one [4]. Recent studies have identified 15 in Arabidopsis [5], 45 in tomato [6], 17 in *A. chinensis* genomes [7], and 11 in sorghum and 21 in *Zea mays*. The evolution and biological functions of WOX have been well-studied in Arabidopsis. WOX members were classified into the modern, intermediate, and ancient clade [8]. AtWOX proteins that belong to the same clade have highly conserved biological properties, whereas WOX members that belong to separate clades have significantly various properties.

Past research has presented that WOX family members are crucial for stem cell conservation and organ formation [9,10,11,12]. The WOX members which belong to intermediate clade were mainly regulated zygote and early embryo development. AtWOX8 is required for normal development of the proembryo, and AtWOX9 plays a key role in controlling the proliferation of meristem cells and furthering embryo growth [13,14]. PaWOX8/9 is the homolog of AtWOX8 and AtWOX9 and may play a conserved role as a regulator of the embryonic foundation [15,16]. CsWOX1 regulates leaf vein development and leaf size via the CsSPL-mediated pathway and/or modulate auxin polar transport and signaling, and may also affect the early reproductive process in cucumber [17,18].

Members of the modern clade contain a specific WUS box (TLXLFPXX), which is also an important component of the WUS gene regulating homeostasis for apical meristem and organ initiation [19,20,21,22]. Except for the cambium stem cell regulator WOX4, members of the WUS clade can partially or fully replace the WUS function in maintenance of shoot apical meristem and floral meristem. Ancient WOX13 and WOX9 are incapable of maintaining stem cells [23,24]. Otherwise, AtWOX11 and AtWOX12, both functionally redundant, have been reported to stimulate the expression of LBD16 and LBD29 and promote the initiation of adventitious root and root hair development [25,26,27]. AtWOX13 belonged to the ancient clade and promoted carpel maturation by inhibiting the activities of JAG/FIL [28]. In addition to controlling the growth of other organs and roots, AtWOX14 also cooperated with AtWOX4 to control the differentiation of vascular meristems [29]. Additionally, it had been shown that AtWOX14 played a stimulating role in the accumulation of biologically-active GA by increasing the production of GA3ox and preventing the breakdown of GA2ox, which in turn promoted the differentiation and lignification of vascular in inflorescence stem cells. *Cis*-elements analysis in the promoter regions found that the faculty of WOX family controlled by plant hormone signaling pathways might be conserved [30,31]. Plants with abnormal stamen and another dysplasia and dwarfed growth were caused by the overexpression of *WOX1*. PRESSED FLOWER (PRS)/WOX3 and WOX1 also took part in the establishment of leaf outgrowth and boundary development downstream of adaxial/abaxial polarity and controlling cell reproduction. [3,32,33,34,35].

WOX family also performs crucial roles in response to various environmental challenges in addition to plant growth and development. For example, eight WOX members (*OsWUS*, *OsWOX3*, *OsWOX4*, *OsWOX5*, *OsWOX9B*, *OsWOX11*, *OsWOX12A*, and *OsWOX12B*) respond to drought, two genes (*OsWOX3 and OsWOX5*) respond to salt, and four genes (*OsWOX5*, *OsWOX9B*, *OsWOX12A*, and *OsWOX12B*) respond to cold stress in rice [36]. Studies have reported that the direct homologous WOX13 in rice responds to various abiotic stresses such as drought, cold and salt [37]. AtWOX6 responds to freezing tolerance through studying the *hos9-1* mutant in Arabidopsis [2]. MdWOX13-1 can directly bind to the promoter of the *MdMnSOD* to increase the callus weight and enhance ROS scavenging under drought stress [38]. *Phvul-WOX-1*, *Phvul-WOX-9* and *Phvul-WOX-16* genes respond to salt stress detected by qRT-PCR [39]. In cucumber, heat and cold shocks reduced the expression of *CsWOX1a, CsWOX3* and *CsWOX4*,and the three *WOX* genes *CsWOX1b*, *CsWOX3*, and *CsWOX5*, respectively, suggesting that which might be involved in the regulation of cucumber stress tolerance [40]. Similarly, a previous study revealed that most of the *WOX* genes can respond to various stresses in cotton [41]. 

Melon is an important economic horticulture crop worldwide. Based on the ovary pubescence, melon can be classified into two subspecies, *C. melo* subsp. *melo* (hereafter as *melo*) and *C. melo* subsp. *agrestis* (hereafter as *agrestis*). The quality and yield of melon were affected by growth environment and development processes. Although the roles of WOX members in regulating many developmental aspects have been exhibited in many crops, there is no characteristic research on WOX members in melon. The updated high-quality genomic sequence of melon provides an opportunity to identify and characterize CmWOX family. In this study, we identified 11 *CmWOX* genes by using the bioinformatics methods based on the melon genome, their phylogeny and conserved domains, investigated their gene expression under cold and drought stress condition, which provides new insights into the evolution and biological functions of *CmWOX* genes in melon.

## 2. Results

### 2.1. Phylogenetic Analysis of the CmWOX Family

**Figure 1:**
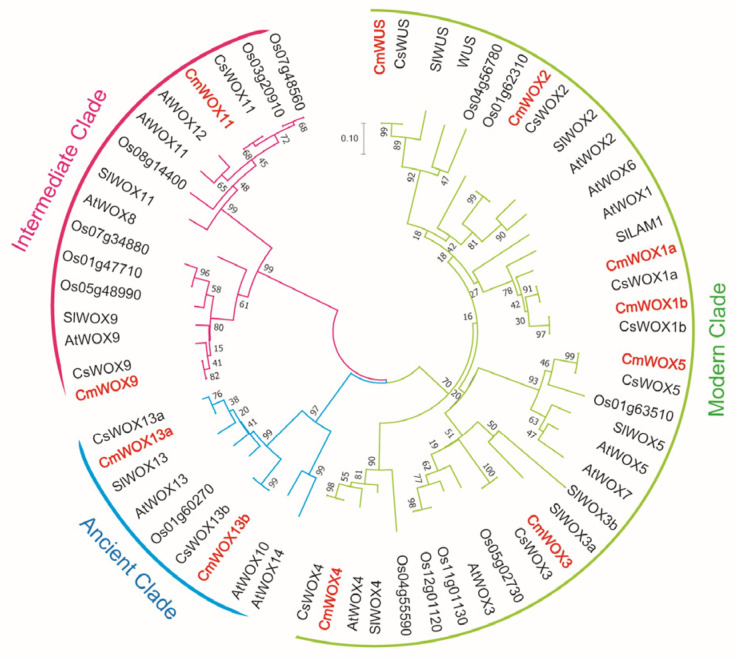
Phylogenetic relationships of the WUSCHEL-related homeobox (WOX). Phylogenetic relationships of the WOX family from Cucumber (Cs), tomato (Sl), Arabidopsis (At), melon (Cm), and rice (Os). The phylogenetic tree was built by MEGA 7.0. The WOX from 5 plants can be divided into 3 groups, each branch is indicated in a specific color.

To explore the evolutionary relationships of WUSCHEL-related homeobox (WOX) family, we performed the phylogenetic analysis of 61 *WOX* genes from Arabidopsis, rice, tomato, cucumber and melon by homologous alignment and MEGA 7.0. Based on the phylogenetic tree, a total of 61 WOX proteins from 5 plants were divided into 3 branches, including the modern clade, the ancient clade, and the intergenic clade. By analyzing paralogous and orthologous relationships of WOX family, we found that most CmWOXs showed closer relationships to CsWOXs, which might be due to the close evolutionary relationship between cucumber and melon. Putative CmWOX family were also identified and classified into the modern clade, intermediate clade, and ancient clade (Figure 1). 

### 2.2. Identification, Description, and Classification of CmWOX Family

To identify WOX family members in melon, a genome-wide analysis was carried out using BLASTp. A total of 11 *CmWOX* genes were identified, consistent with the previous study in identifying CsWOX family [42], and then we named the *CmWOX* genes after the CsWOX family according to the phylogenetic and evolutionary relationship. The phylogenetic tree included the WOX family of melon (Cm), cucumber (Cs), tomato (Sl), Arabidopsis (At) and rice (Os). The coding sequences of these genes range from 519 to 1140 bp (Base Pair) in the melon (DHL92) v4 Genome. The amino acid (AA) number ranges from 172 to 385, and the molecular weights between 14.66 and 43.64 kDa and isoelectric point (pI) values from 5.23 to 10.312, the number of amino acids of the whole family are from 172 to 385 (Table 1). Although CmWOXs belong to a family, the basic information varies greatly.

### 2.3. The Chromosomal Localization of CmWOX Genes

**Figure 2:**
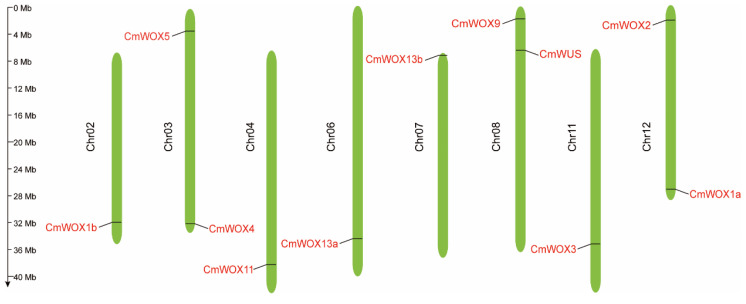
Distribution of *CmWOX* genes on the chromosome in melon. Total 11 *CmWOX* genes are located in assembled chromosome. The number of chromosome was noted on the left side of each chromosome. The bar scale on the left is in Mega base (Mb).

The chromosomal location of *CmWOX* genes was analyzed through the MapGene2Chrom online website [43], and the result exhibited that *CmWOX* genes were irregularly distributed on eight chromosomes (Figure 2; Supplementary Table S2). There was one *CmWOX* gene on chromosome 2 (*CmWOX1b*), 4 (*CmWOX11*), 6 (*CmWOX13a*), 7 (*CmWOX13b*), 11 (*CmWOX3*), and two *CmWOX* genes on chromosome 3 (*CmWOX5* and *CmWOX4*), 8 (*CmWOX9* and *CmWUS*), and 12 (*CmWOX1a, CmWOX2*), and no *CmWOX* genes were discovered on other four chromosomes of melon. Gene duplication events were investigated among the *CmWOX* genes, no segment duplication pairs were identified in this family. 

### 2.4. Gene Structure Analysis of CmWOX Genes

**Figure 3:**
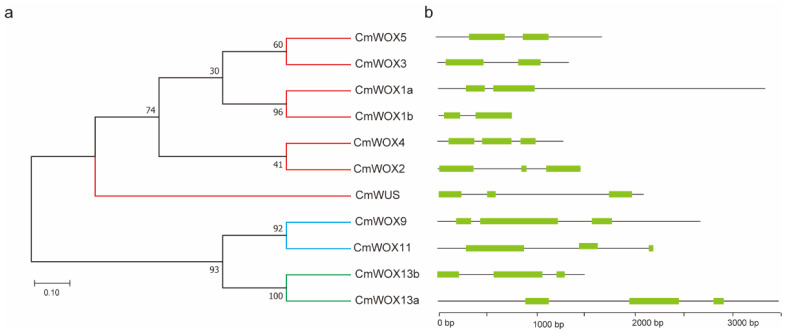
Phylogenetic analysis and gene structure of *CmWOXs.* (**a**) MEGA 7.0 was used to build the phylogenetic tree of 11 CmWOX proteins and divided them into the modern clade, the intermediate clade, and the ancient clade, each clade was indicated in red, blue and green line, respectively. (**b**) Exon-intron structures of *CmWOX* genes were drawn by the Gene Structure Display Server, Green boxes indicated exons and black lines indicated introns.

Gene structure is important for determining the relationship between genomic evolution and functional differentiation of multi-genic family members. MEGA 7.0 was used to build the phylogenetic tree of 11 CmWOX proteins. CmWOX1a, CmWOX1b, CmWOX3, CmWOX4, and CmWOX5 were divided into the modern clade; CmWOX9 and CmWOX11 were divided into the intermediate clade; and CmWOX13a and CmWOX13b were divided into ancient clade. In order to investigate the structural diversity of *CmWOX* genes, the exon-intron map was constructed according to the genome and coding sequences of *CmWOX* genes (Figure 3a). The number of introns in *CmWOXs* varies from one to two. Combined with the results of phylogenetic trees, the modern/WUS group with 1-2 intron insertions can be further divided into three sub-clades. *CmWOX5*, *CmWOX3*, *CmWOX1a*, and *CmWOX1b* contained 1 intron. The other two members, *CmWOX4* and *CmWOX2*, had two introns. Both intermediate (*CmWOX9* and *CmWOX11*) and ancient group (*CmWOX13a* and *CmWOX13b*) contained two introns. Seven members of the *CmWOX* genes contain 3 exons, and 4 *CmWOX* genes possess 2 exons (Figure 3b). There was a big difference among *CmWOX* genes in numbers and lengths of exon and intron, which suggested that CmWOX family may play a specific or redundant role in evolution.

### 2.5. The Conserved Motifs Analysis of CmWOX Proteins

**Figure 4:**
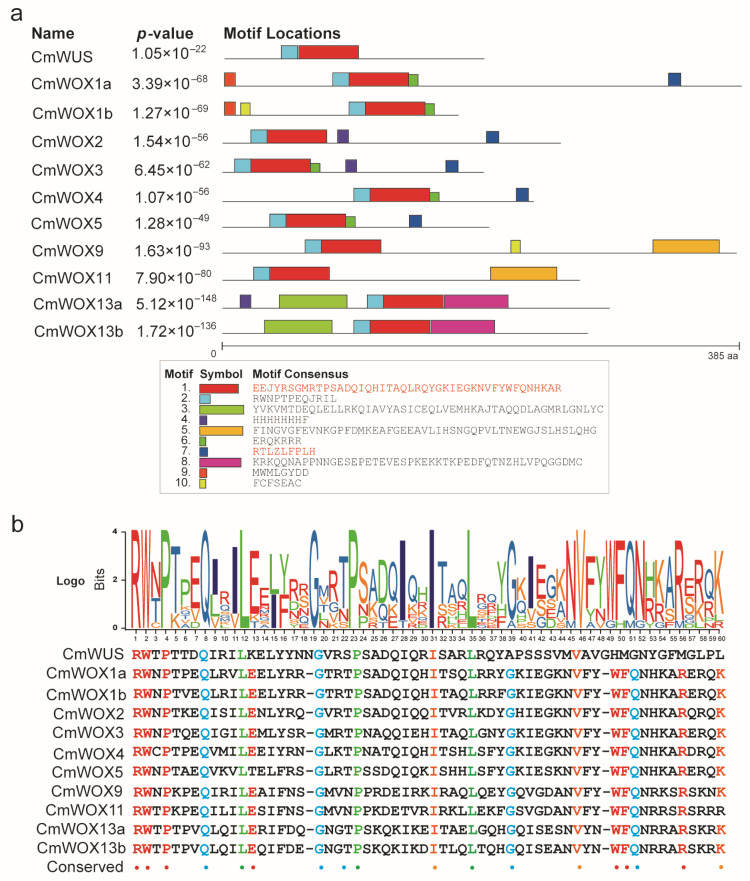
The conserved motifs and homeodomain of CmWOX family. (**a**) The conserved motif 1 to motif 10 of CmWOX proteins were highlighted with different colored boxes. Motif 1 and motif 7 were homeodomain and WUS motif, respectively. (**b**) The seq-logo of conserved domains was analyzed using the SeqLogo program of TBtools. Different colors indicated highly conserved residues, which are R, W, P, Q, G, I, L, W, and F.

The conserved motifs of WOX members played a key role in regulating plant cell specificity, and the size of flower meristem and worked as a transcript repressor. CmWOX proteins were examined to find the motifs through PlantCARE, MEME suite and TBtools [44,45]. A total number of 10 conserved motifs were prefigured in CmWOX proteins, and the amino acid number were ranged from 15 to 50. The results revealed that groups classified by phylogenetic analysis contained similar motif compositions, but there were some differences between the different subgroups. Motifs 1 and 2 were existing in all the CmWOX proteins, and the motif 2 was the conserved homeodomain (Figure 4a). Motif 7 (WUS motif) only existed in modern clade, and was located in CmWUS, CmWOX1a, CmWOX2, CmWOX3, CmWOX4, and CmWOX5, whereas motifs 3 and 8 only existed in ancient CmWOX members. Motif 5 only existed in intergenic members. Motif 6 presented in CmWOX3, CmWOX4, CmWOX5, CmWOX1a, and CmWOX1b. The motif 4 presented in CmWOX13a, CmWOX2, and CmWOX5. Motif 9 presented in CmWOX1a and CmWOX1b, and the motif 10 presented in CmWOX9 and CmWOX1b. The homeobox seq-logo of the CmWOX was analyzed using the SeqLogo program (TBtools (v1.120)). R, W, P, Q, G, I, L, W and F residues were highly conserved in CmWOX proteins (Figure 4b). To some degree, these specific and conserved motifs of CmWOX proteins may lead to different functions or functional redundancy in plant growth and development, which need to be further explored.

### 2.6. The Cis-Elements Analysis in Promoter Regions of CmWOX Genes

**Figure 5:**
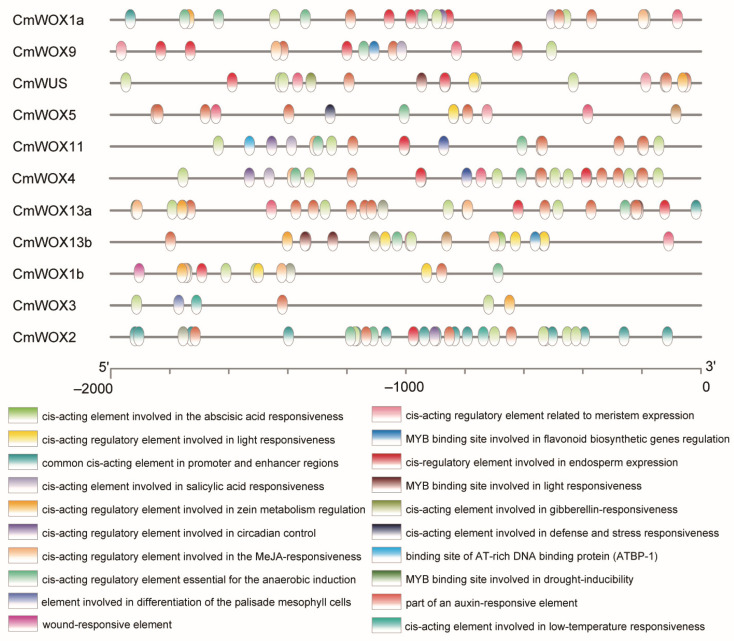
Predicted *cis*-elements in promoter regions of *CmWOX* genes. A promoter region of about 2-kb upstream of *CmWOX* genes are showed potential *cis*-elements, particularly those associated with stress responses (such as light induction, low temperature, and anaerobic induction) and plant hormones (such as auxin and gibberellin). Different *cis*-elements are stated with different colors.

*Cis*-elements in the promoter region of the *WOX* genes were involved in affecting plant growth and development through regulating expression of target gene. It is meaningful to analyze the *cis*-acting elements of the *CmWOX* family in their promoter regions. The analysis results revealed that many *cis*-motifs might be taken part in response to biotic and abiotic stresses, such as low temperature, cold, and photo stresses (Figure 5; Supplementary Table S3). Abiotic stress-responsive *cis*-elements were found in the majority regions of the *CmWOX* promoter. However, drought and low-temperature responsive elements were only detected in the promoter region of *CmWOX13a*, *CmWOX3*, and *CmWOX2*, respectively. Additionally, we discovered light-sensitive elements in nearly all the promoter regions of the *CmWOX* genes, suggesting that light signaling may take part in controlling the expression of *CmWOX*. Six out of the eleven *CmWOX* promoters had MeJA-responsive elements. Abscisic acid-responsive elements were identified in seven *CmWOX* promoters, whereas auxin-responsive elements were only found in the *CmWOX13a* promoter. These results indicated that CmWOX members may response to stresses and regulate the plant growth and development crossed with plant hormone. 

### 2.7. Transcriptome Data Analysis under Cold and Drought Treatments

**Figure 6:**
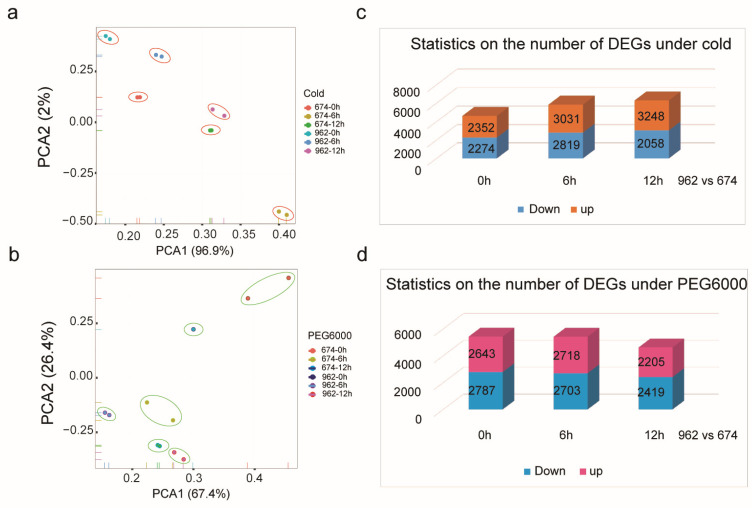
Principle component analysis (PCA) and statistics on the number of DEGs between ‘674’ and ‘962’. (**a**,**b**) Principle component analysis of samples in ‘674’ and ‘962’under cold and PEG6000 treatment, respectively. (**c**,**d**) Statistics on the number of DEGs in ‘674’ and ‘962’ under cold and PEG6000 treatment. The number of differential expressed genes is counted at three different time points.

Research has reported that *CmWOX* genes are involved in abiotic stresses in horticultural crops, such as tomato and cucumber. To investigate the roles of the *CmWOXs* under cold and drought condition, we analyzed the gene expression of this family in the leaves of melon seedlings. After analysing of the *cis*-element in the promoter regions of *CmWOXs*, leaves in the seedlings stage were subjected to RNA-seq gene expression analysis under cold and PEG6000 treatments. Differential genes expression levels were calculated by FPKM, the expression data of all sequenced samples were then subjected to principal component analysis (PCA) and DEGs analysis (Figure 6; Supplementary Table S4). The PCA of the expressed biographies of the 12 libraries (6 samples with 2 replicates each, Figure 6a,b) revealed that samples collected at different hours can be obviously different. PCA1 explained 96.9% and 67.4% of the variance for cold and PEG6000, respectively. Thus, the results were verified to be highly dependable and acceptable for further analyses. Under the cold treatment, there were 4626 DEGs between ‘962-0 h’ and ‘674-0 h’, of which 2352 DEGs were up-regulated and 2274 DEGs were down-regulated. A total of 5850 DEGs between ‘962-6 h’ and ‘674-6 h’, of which 3031 DEGs were up-regulated and 2819 were down-regulated. A total of 6306 DEGs between ‘962-12 h’ and ‘674-12 h’, of which 3248 DEGs were up-regulated and 3058 were down-regulated, which are higher than that at 0 h and 6 h (Figure 6c). Moreover, under the PEG6000 treatment, there were 5430 DEGs between ‘962-0 h’ and ‘674-0 h’, of which 2718 DEGs were up-regulated and 2703 were down-regulated. The number of DEGs between ‘962-6 h’ and ‘674-6 h’ is similar to the sample at 0 h. While 4624 DEGs between ‘962-12 h’ and ‘674-12 h’, of which 2205 DEGs were up-regulated and 2419 were down-regulated, which are lower than that at 0 h and 6 h (Figure 6d). The number of DEGs under cold and PEG6000 treatment differed considerably between in *melo* and *agrestis*. Furthermore, studying the functions of the differential expressed genes among *CmWOXs* will help us to understand the molecular mechanism of melon response to stresses.

### 2.8. The Expression Pattern of CmWOX Gene in Melon Leaves under Cold and PEG6000 Treatments

**Figure 7:**
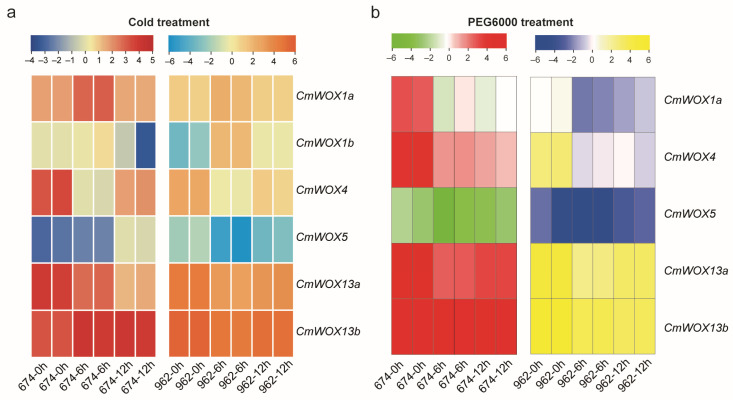
The heat map of *CmWOX* genes under cold and drought stresses. The heat map of the expression of typical *CmWOX* genes in melon. (**a**) The transcriptomes heat map of ‘674’ and ‘962’ *CmWOX* family under low-temperature treatment at 0 h, 6 h, and 12 h. (**b**) The transcriptomes heat map of ‘674’ and ‘962’ *CmWOX* family under PEG6000 treatment at 0 h, 6 h, and 12 h. Color scale represents fold changes (log2 fold change). Blue and green colors indicate low expression level, red and yellow color indicate high expression level, respectively. The data were processed from 2 replicates of transcriptomic data at different treatment times.

Transcriptome data analysis showed that the expression level of *CmWOX1a*, *CmWOX1b*, *CmWOX4*, *CmWOX5*, *CmWOX13a*, and *CmWOX13b* changed with the treating time. While no obvious changes in transcript levels were observed in the *CmWUS*, *CmWOX2*, *CmWOX3*, *CmWOX9*, and *CmWOX11* under cold and 20% PEG6000 condition. *CmWOX13a* and *CmWOX13b* were constitutively expressed in both ‘674’ and ‘962’. The log2 (fold change) of *CmWOX13a* and *CmWOX13b* were higher than other genes (Figure 7a,b; Supplementary Tables S4 and S5). The above results suggested that some CmWOX members were involved in abiotic stress response during melon growth and development. 

### 2.9. The Expression Level of CmWOX Genes under Cold and PEG6000 Treatments

**Figure 8:**
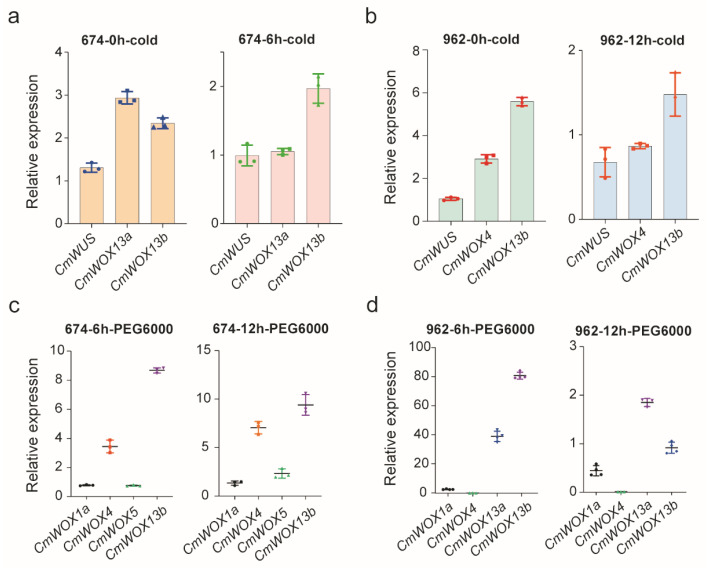
The expression level of *CmWOX* genes under cold and drought stresses. (**a**) QRT-PCR verified the expression of *CmWUS*, *CmWOX13a*, and *CmWOX13b* at 0 h and 6 h under cold treatment in ‘674’. (**b**) QRT-PCR verified the expression of *CmWUS*, *CmWOX4*, and *CmWOX13b* at 0 h and 12 h under cold treatment in ‘962’. (**c**) QRT-PCR verified the expression of *CmWOX1a*, *CmWOX4*, *CmWOX5*, and *CmWOX13b* at 6 h and 12 h under PEG6000 treatment in ‘674’. (**d**) QRT-PCR verified the expression of *CmWOX1a*, *CmWOX4, CmWOX13a*, and *CmWOX13b* at 6 h and 12 h under PEG6000 treatment in ‘962’. *CmACTIN* was used as an internal control, and three or four biological replicates were calculated, error bars indicate the standard error of the mean. The different colors of symbols circle, square, and arrow denote different genes.

To confirm the expression level of *CmWOX* genes acquired by RNA-seq data, qRT-PCR assay was conducted. According to the log2 (value) analysis, four genes, including one orthologous pair, were supposed to be highly expressed under cold stress. The expression pattern of the orthologous genes (*CmWOX13a* and *CmWOX13b*) were detected by qRT-PCR in leaves of ‘674’ and ‘962’. The results showed a similar tendency with the RNA-seq data (Figure 8a,b). The expression of *CmWUS* was undetected at 0 h, 6 h, and 12 h under cold and drought treatments in both ‘674’ and ‘962’. These results revealed that *CmWUS* did not response to abiotic stresses, and may play a conserved role in controlling the proliferation of plant shoot and root stem cells. The expression of *CmWOX4* was decreased both under cold and drought treatments in ‘962’. However, the expression levels of *CmWOX13a* and *CmWOX13b* were high in both ‘674’ and ‘962’ under cold and drought stresses, suggesting that which might be the key players and potential candidate genes in response progress to stresses in melon (Figure 8c,d). 

## 3. Discussion

Transcript initiation and gene expression regulation are significantly influenced by transcription factors. The *WOX* gene family encodes transcription factors unique to plants that are important for maintaining shoot and root stem cell homeostasis, developing tissues, and growing organs [11,22]. The *WOX* gene family has been identified in many plants, and so far, detailed information on this family in melon has not been uncovered. The updating and optimized genome of melon provides a chance for us to systematically analyze the *CmWOX* family. In this study, we identified and characterized 11 *CmWOXs* through genome-wide analysis. The WOX family members in melon are less than that in Arabidopsis, tomato, rice, and maize, the same number as in cucumber and sorghum [12,42]. Phylogenetic analysis showed that CmWOX proteins had a closer relationship with that in cucumber (Figure 1; Supplementary Table S1), which is consistent with the discovery that melon and cucumber are most homologous in Cucubitaceae [46].

Although the CmWOX family has a conservative homeodomain and WUS domain based on the conserved motif analysis, each member also has their distinctive motifs. The genomic structure was highly varied even within the same family, according to homology analyses (Figure 4). Those findings implied that the *CmWOX* gene family may have both functional redundancy and specific regulatory effects. Moreover, various studies found that the *WOX* genes respond to plant hormones and abiotic stresses, suggesting that they have important functions in regulating plant growth and development [25,35,37,47]. According to the results analyzed by MEME and TBtools, we discovered that the promoter regions of the *CmWOX1a*, *CmWOX1b*, *CmWOX13a*, and *CmWOX13b* contain 2-3 *cis*-elements responded to auxin, gibberellin, and jasmonic acid. Furthermore, we discovered *cis*-elements that are responsive to auxin in the promoter regions of the *CmWOX2* and *CmWOX4*, responsive to jasmonic acid in that of the *CmWOX9* and *CmWOX11*, and responsive to GA in the case of the *CmWUS*, *CmWOX4*, *CmWOX5*, and *CmWOX9* (Figure 5). According to the aforementioned results, *CmWOX* genes may participate in the process of plant hormone signal transduction and be crucial in controlling the initiation and development of plant organs.

A previous study revealed that the two subspecies of melon, *melo* and *agrestis*, were domesticated independently [48]. The independent domestications might greatly influence gene duplication, differentiation, expression, and function finally resulting in various characteristics between *melo* and *agrestis*. According to transcriptome databases, the expression of genes varies in ‘674’ and ‘962’. The different expressed gene number (DEGs) in *melo* and *agrestis* under the two treatments was different. The majority of them were DNA-binding proteins that participate in abiotic conditions, stimuli and biological development, and played a role in regulating the transcriptional levels of downstream genes (Figure 6). Many studies have reported that (CBF/DREB1)- and ABA -related genes were involved in cold and drought stresses [49,50]. Therefore, we detected that the *CmPYR*, *CmPP2C-1*, and *CmPP2C-2* are ABA- related genes, and *CmDREB2A*-like, *CmERF039*-like, and *CmDREB3*-like are *CmDREB* family members in melon according to the homologous proteins alignment. The transcript level of the *CmPYR*, *CmPP2C-1* and *CmPP2C-2* were up-regulated under PEG6000 treatment (Figure S1a). *CmERF039*-like respond to cold, which was up-regulated in ‘674’ and down-regulated in ‘962’ under cold stress, while both of *CmDREB2A*-like and *CmDREB3-like* were up-regulated in ‘674’ and ‘962’ under cold treatment in the transcriptome data (Figure S1b, Supplementary Table S8). There were different genes responding to abiotic stress compared with cucumber. Several *CmWOX* genes were expressed in leaves, except for *CmWUS*, *CmWOX2*, *CmWOX3*, *CmWOX9*, or *CmWOX11*. Unlike the *CsWOX1a* and *CsWOX5* response to drought treatment, there was no difference on transcript level in melon with the same treatment. Although the evolution relationship between melon and cucumber are close, the functions of WOX family members in two species are shown differently. The expression levels in leaves under cold stress were higher than those of other family members for the two *CmWOX13a* and *CmWOX13b* members, indicating that they had a regulatory influence on melon stress response (Figure 7), which is consistent with the previous study in cucumber [40,42,51]. These findings demonstrated the divergence of *CmWOX* genes as well as the pattern of expression under cold in two melon subspecies. However, it is necessary to study the biological function of the *CmWOX* genes in the future.

## 4. Materials and Methods

### 4.1. Plant Materials and Growth Conditions

Melon seeds of ‘674’ (*C. melo subsp. melo*, derived from Japan) and ‘962’ (*C. melo subsp. agrestis*, derived from China) were provided by obtained from the National Mid-term Genebank, soaked at 55 °C for 3 h first and then transferred to the dark 28 °C incubator for germinating. Seedlings grew in a growth chamber under long-light condition (16 h light/8 h dark cycle with 100 μM photons m^−2^ s^−1^ at 28 °C for 2 weeks). Two-week-old seedlings were treated by 20% (*w*/*v*) PEG6000 and 8 °C in the chamber. Leaves were collected after time intervals at 0, 6, and 12 h, and non-treated seedlings as control. Leaf samples were frozen immediately in liquid nitrogen and subsequently stored at ultra-low temperature (−80 °C) freezer for RNA extraction and subsequent for the expression level analysis.

### 4.2. Identification, Characteristics, and Phylogenetic Analysis of CmWOX Genes

The identification and analysis of melon WOX genes were performed using genome sequence data of melon (*Cucumis melo* L., cv. DHL92 v4 Genome) deposited at the Cucurbit Genomics Data website (http://cucurbitgenomics.org/v2/organism/23; accessed on 24 March 2023) [52]. CmWOX homologous protein sequences of Arabidopsis, tomato and rice were downloaded from the genome database and used as query sequences to search for putative counterparts in melon by using BlastP. All *CmWOX* genes were named according to their homologous relationship with that in cucumber. The multiple sequence alignment for the full-length amino acid sequences of AtWOX, CsWOX, OsWOX, and SlWOX family members were chosen [53]. The phylogenetic tree was constructed using the neighbor-joining (NJ) method with settings of pairwise deletion, Poisson correction, and 1000 bootstraps analysis for CmWOX proteins. The total 11 amino acid sequences of CmWOX family were used to be analyzed. All positions containing gaps and missing data were eliminated. There were a total of 86 positions in the final dataset. Evolutionary analyses were conducted through MEGA7 (Version 7.0), and the distributions of *CmWOXs* on chromosomal were rendered using MapGene2 Chrome (http://mg2c.iask.in/mg2c_v2.1/; accessed on 24 March 2023) [43].

### 4.3. Gene Structure and Motif Analysis 

The genomic and CDS sequences of *CmWOX* genes were from the melon genome database, and the gene structures were analyzed and visualized according to the genome sequence by TBtools. The conserved motifs of CmWOX amino acids were identified with MEME 5.5 (https://meme-suite.org/meme/doc/meme.html; accessed on 24 March 2023). Totally, 10 conserved motifs were identified for the melon WOXs and designated as motif 1 to motif 10. 

### 4.4. Putative Promoter Region Analysis of CmWOX Genes

To identify *cis*-elements in promoter regions, the 2.0 kb sequences upstream of the translational start site (TSS) of melon WOX genes were downloaded from the Cucurbit Genomics Data website. Conserved motifs were analyzed with the online tool PlantCARE (http://bioinformatics.psb.ugent.Be/webtools/plantcare/html/; accessed on 24 March 2023), visualized these elements through TBtools [44,54].

### 4.5. RNA Extracting and RNA-seq Data Analysis

Three-week-old melon seedlings of ‘674’ and ‘962’ were grown in a growth chamber and 8 °C for cold treatment for 12 h at normal light/dark condition. Leaves were collected at 0, 6 and 12 h after treatment. Total RNA was extracted from the treated leaves using an FT-plant RNA Isolation kit (NS-26, NONAZYME). The sequencing library was constructed using an Ultra RNA sample preparation kit and then sequenced using a Novaseq-S4-150PE according to the standard method (Berry Genomics, Fuzhou, China). Total reads were mapped to the DHL92 genome (V4.0). Differentially expressed genes were identified using Cuffdiff with default criteria (log2 (fold change) ≥ 1) and adjusted false discovery rate (*p* value < 0.05). Two independent biological replicates were used for the RNA-sequencing analysis.

### 4.6. Gene Expression and Real-Time PCR Analysis

The expression patterns of *CmWOX* genes were graphically represented in a heat map by cluster analysis using TBtools software (V1.120). Primers were designed for qRT-PCR verification of the selected *CmWOX* genes through NCBI, and *CmACTIN* gene was used as an internal control. The primers for qRT-PCR were synthesized by Sangon Biotech (Zhengzhou, China); the details are shown in Supplemental Table S7. Reverse transcription PCR (RT-PCR) was carried out with an All-in-one mix kit (Bioman, Shanghai, China). QRT-PCR detections for the target genes were performed with the SYBR Green FAST Mixture qPCR kit (GenStar, Shenzhen City, China) through the LightCycler480 real-time PCR detection System (Roche Diagnostics, Indianapolis, IN, USA), following by the PCR program of 95 °C for 30 s, 40 cycles of 95 °C for 15 s and 60 °C for 15 s, and finally 72 °C for 30 s. Relative expression levels of genes were measured through the 2^−ΔΔCT^ method [55,56,57].

### 4.7. Statistical Analysis

A minimum of three biological replicates were used per experiment. Results are provided as means ± SD using GraphPad Prism 8.

## 5. Conclusions

In this study, a genome-wide analysis of *CmWOX* genes was conducted in melon. A total of 11 *CmWOX* genes were identified and described, and were divided into three subclades according to phylogenetic and structural analysis. Protein sequence alignment showed that CmWOX family had a typical homeodomain and conserved motifs. There are many *cis*-elements in the promoter region of *CmWOXs* which were respond to cold, drought and plant hormone. *CmWOX* genes displayed different expression patterns under cold and drought stresses in *melo* and *agrestis*. *CmWOX13a* and *CmWOX13b* were expressed at a higher level in comparison with other *CmWOX* genes. RNA-seq and qRT-PCR results imply that *CmWOX4* may play a role in abiotic processes during plant development. Taken together, our work provides insights into the homology and characteristics of the *CmWOX* genes, which helps further research on their biological functions in tolerance.

## Figures and Tables

**Table 1:** Identification and characteristics of CmWOX family genes.

Name	Clade	CDS (bp)	AA	MW (kDa)	PI
*CmWUS*	Modern Clade	576	195	21.18	10.228
*CmWOX1a*	Modern Clade	1146	385	43.13	5.699
*CmWOX1b*	Modern Clade	519	172	20.03	10.312
*CmWOX2*	Modern Clade	750	249	28.21	9.211
*CmWOX3*	Modern Clade	579	196	22.12	6.424
*CmWOX4*	Modern Clade	690	233	26.02	8.56
*CmWOX5*	Modern Clade	591	196	22.21	8.637
*CmWOX9*	Intermediate Clade	1140	383	41.57	7.688
*CmWOX11*	Intermediate Clade	792	271	28.09	5.665
*CmWOX13a*	Ancient Clade	858	293	32.51	6.204
*CmWOX13b*	Ancient Clade	810	277	30.63	5.23

## Data Availability

The BioProject accession number of the RNA-seq raw data of ‘674’ and ‘962’ under cold and PEG6000 treatments is PRJNA952866.

## Supplementary Materials

The following supporting information can be downloaded at: https://www.mdpi.com/article/10.3390/ijms241512326/s1.
