# Supplementary material for: Genome-Wide Identification and Characterization Analysis of WUSCHEL-Related Homeobox Family in Melon (Cucumis melo L.)
Source: Int J Mol Sci. 2023 Aug 1;24(15):12326. doi: 10.3390/ijms241512326 (PMC10419029; doi:10.3390/ijms241512326)
Supplement: Supplementary file 1 [file ijms-24-12326-s001.zip › Supplemental figure---23.07.26.pdf]

Supplemental figure:

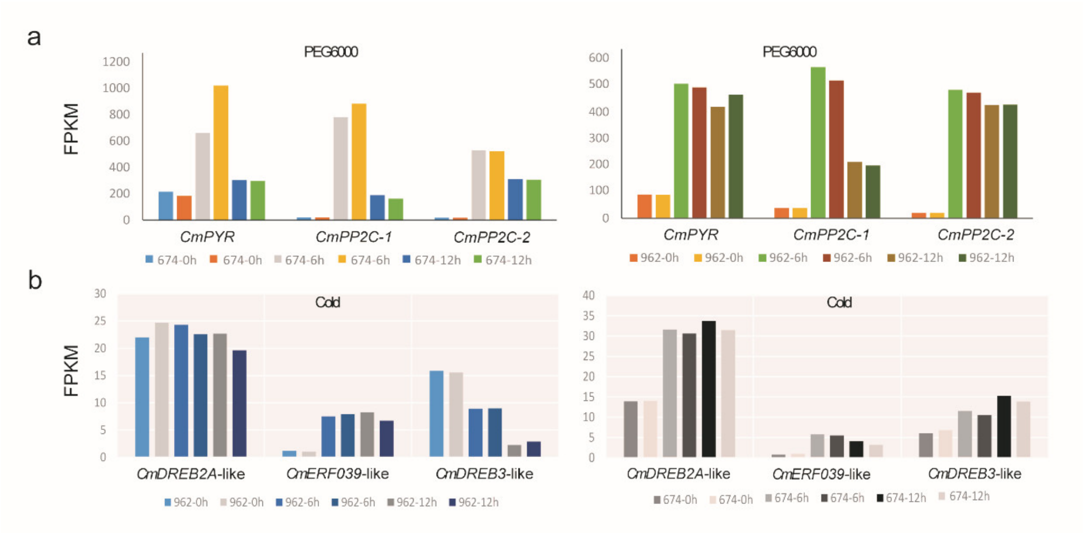

Figure S1. Transcript levels of ABA related genes and *CmDREB* -like genes.

(a) Transcript levels of ABA -related genes under PEG6000 of '674' and '962'. **(b)** Transcript levels of *CmDREB* -like genes under cold of '674' and '962'.
